# Diesel exhaust particle exposure *in vitro* impacts T lymphocyte phenotype and function

**DOI:** 10.1186/s12989-014-0074-0

**Published:** 2014-12-14

**Authors:** Marina Pierdominici, Angela Maselli, Serena Cecchetti, Antonella Tinari, Arianna Mastrofrancesco, Michela Alfè, Valentina Gargiulo, Carlo Beatrice, Gabriele Di Blasio, Giulia Carpinelli, Elena Ortona, Antonello Giovannetti, Silvana Fiorito

**Affiliations:** Department of Cell Biology and Neurosciences, Istituto Superiore di Sanità, Rome, Italy; Department of Technology and Health, Istituto Superiore di Sanità, Rome, Italy; San Gallicano Dermatologic Institute, IRCCS-IFO, Laboratory of Cutaneous Physiopathology and Integrated Center of Metabolomics Research, Rome, Italy; Istituto di Ricerche sulla Combustione (IRC), CNR- Naples, Italy; Istituto Motori (IM), CNR-Naples, Italy; Istituto San Raffaele Sulmona, Sulmona, Italy; Department of Clinical Medicine, Division of Clinical Immunology, Sapienza University of Rome, Rome, Italy; Institute of Translational Pharmacology, CNR-Rome, Italy; Research Center for Nanotechnologies applied to Engineering-CNIS, Rome, Italy

**Keywords:** Air pollution, DEP, T lymphocytes, Autophagy, Mitochondria, IL-2

## Abstract

**Background:**

Diesel exhaust particles (DEP) are major constituents of ambient air pollution and their adverse health effect is an area of intensive investigations. With respect to the immune system, DEP have attracted significant research attention as a factor that could influence allergic diseases interfering with cytokine production and chemokine expression. With this exception, scant data are available on the impact of DEP on lymphocyte homeostasis. Here, the effects of nanoparticles from Euro 4 (E4) and Euro 5 (E5) light duty diesel engines on the phenotype and function of T lymphocytes from healthy donors were evaluated.

**Methods:**

T lymphocytes were isolated from peripheral blood obtained from healthy volunteers and subsequently stimulated with different concentration (from 0.15 to 60 μg/ml) and at different time points (from 24 h to 9 days) of either E4 or E5 particles. Immunological parameters, including apoptosis, autophagy, proliferation levels, mitochondrial function, expression of activation markers and cytokine production were evaluated by cellular and molecular analyses.

**Results:**

DEP exposure caused a pronounced autophagic-lysosomal blockade, thus interfering with a key mechanism involved in the maintaining of T cell homeostasis. Moreover, DEP decreased mitochondrial membrane potential but, unexpectedly, this effect did not result in changes of the apoptosis and/or necrosis levels, as well as of intracellular content of adenosine triphosphate (ATP). Finally, a down-regulation of the expression of the alpha chain of the interleukin (IL)-2 receptor (i.e., the CD25 molecule) as well as an abnormal Th1 cytokine expression profile (i.e., a decrease of IL-2 and interferon (IFN)-γ production) were observed after DEP exposure. No differences between the two compounds were detected in all studied parameters.

**Conclusions:**

Overall, our data identify functional and phenotypic T lymphocyte parameters as relevant targets for DEP cytotoxicity, whose impairment could be detrimental, at least in the long run, for human health, favouring the development or the progression of diseases such as autoimmunity and cancer.

**Electronic supplementary material:**

The online version of this article (doi:10.1186/s12989-014-0074-0) contains supplementary material, which is available to authorized users.

## Background

Particulate matter in air pollution is associated with adverse health effects such as asthma and cardiovascular diseases as well as lung cancer mortality [1-4]. Diesel exhaust particles (DEP) emitted by diesel engines consist of fine particles (particulate matter with an aerodynamic diameter ≤ 2.5 μm) including a high number of ultrafine particles (< 0.1 μm diameter). They are composed of a center core of elemental carbon (80%) and adsorbed organic compounds, including polycyclic aromatic hydrocarbons (PAH) and nitro-PAH, and small amounts of sulfate, nitrate, metals, and other trace elements. All these compounds are considered to be of great toxicological importance. The small size of DEP makes them highly respirable, thus having the potential to reach the deep lung and to translocate to the bloodstream although this latter still remains a debated issue [2,5-10]. In particular, it has been suggested that ultrafine carbon particles, after deposition in the lung, largely escape alveolar macrophage surveillance and gain access to the pulmonary interstitium. From this site, a further translocation of the ultrafine particles to the blood circulation via lymphatic channels or directly via the endothelium could take place [8,9].

A series of studies *in vivo* revealed that DEP exposure has remarkable effects on the immune system: pre- and postnatal animal exposures to DEP decrease the weight of the thymus and spleen, accelerate the production of IgE against pollen, increase allergic susceptibility, alter inflammatory indices in the lung, and increase airway hyperesponsiveness [11,12]. These findings in animal models have been partially confirmed in *in vitro* and *in vivo* human studies, and the largest literature in this regard has looked at the link between DEP exposure and allergic diseases. In fact, it has been demonstrated that DEP exposure can both exacerbate existing allergic diseases and cause allergic sensitization by promoting a Th2 cytokine profile [12-24]. The precise mechanism by which DEP exposure promotes allergic responses is not entirely clear, although oxidant activity of the adsorbed PAH, rather than properties specific to the carbon core, appears to be involved. With the exception of these studies regarding cytokine production, scant data are available on the impact of DEP on lymphocyte phenotype and function. This topic has substantial importance in light of evidence that aberrant lymphocyte homeostasis can result in several diseases including autoimmune, allergic and even neoplastic diseases. In one study, chronic *in vitro* exposure of T lymphocytes to DEP-PHA increased T cell activation marker expression and proliferation in asthmatics but not in controls [19]. More recently, Vattanasit *et al.* [25] demonstrated that reactive oxygen species generation and oxidative DNA damage were induced by DEP in both lymphoblasts and lung cells suggesting that lymphocytes could be used as a surrogate to assess DEP-dependent responses in the lung. No data are currently available on the effects of DEP on T cell fate in terms of apoptosis or autophagy. This latter is a lysosome-mediated catabolic process that allows cells to degrade unwanted cytoplasmic constituents and recycle nutrients [26], and it has been recently emerged as a key parameter, in addition to apoptosis [27], in the maintaining of lymphocyte homeostasis [28-31].

In the last years, all major automobile companies, in order to decrease the dangerous effects of the environmental pollution deriving from DEP on human health, produced and put into the market diesel engines at lower particle emission rate than in the past as well as filters for soot particles. Nevertheless, these strategies neglected the question of how soot quality, more than quantity, may change its effect on human health. Our previous findings demonstrated that carbon based nanoparticles from a low emission diesel engine (Euro 4, E4) are more toxic against human macrophage and skin cells than the older diesel engine black soot (BS), highlighting how low-emission engine soot has a higher toxic potential per unit mass than the soot produced from an older engine [32,33]. In the present study, the impact of nanoparticles from E4 and Euro 5 (E5) light duty diesel engines on the phenotype and function of circulating T lymphocytes from healthy donors was evaluated in order to assess whether environmental nanoparticulate is able to interfere with T cell homeostasis, thus favouring, at least on a susceptible background, the development of disorders associated with abnormal lymphocyte homeostasis. To this aim, different immunological parameters including apoptosis, autophagy, proliferation levels, mitochondrial function, expression of activation markers and cytokine production were evaluated.

## Results

### E4 and E5 chemical-physical features

A detailed chemical and structural characterization of E4 and E5 soot was reported previously [34]. Briefly, E5 soot aerodynamic diameter, measured by a differential mobility spectrometer (DMS), was observed to be slightly larger (90 ± 5 nm) than that of E4 soot (80 ± 5 nm). This finding was confirmed by the evaluation of the hydrodynamic diameter measured using dynamic light scattering (DLS, 115 ± 5 nm and 95 ± 5 nm, for E5 and E4 respectively). Both E4 and E5 soots consisted of irregularly shaped compact aggregates of almost spherical primary particles (15–20 nm). Infrared and UV–vis absorption spectroscopy indicated that the samples exhibited predominantly sp2-hybridization, indicative of the presence of highly conjugated systems [35]. The highly conjugated systems (graphene layers) were tightly connected each other in a compact aggregate constituting the center core of elemental carbon with well-defined morphological features, as imaged by high resolution transmission electron microscopy (HRTEM). The soot was pretreated (as described in soot sampling and pre-treatment paragraph) in order to remove all non-covalently bound molecules adsorbed on its surface. The presence of oxygen functional groups (mainly C = O) was also detected. Although E4 and E5 soots appeared quite similar in terms of surface functionalities, the graphitization degree was slightly more pronounced in the E5 soot (73%), indicating a lower presence of defective sites (bent graphene layers, oxygenated sites) with respect to E4 soot (69%). Even though non-specific interactions (i.e., hydrophobic, van der Waals interactions) arose between the particles when sampled on the filter, nano- and micro-structures (primary particles dimension, aggregate size, particle size distribution) and surface chemical-physical properties resulted unaffected. As concerns the size distribution of the particles, a powerful solvent as N-metyl pirrolidinone (NMP) was able to disperse the particles in a colloidal stable suspension demonstrating the non-covalent nature of the soot aggregates [35,36]. Moreover, DLS performed on NMP soot suspensions demonstrated that the aggregate diameter of the soot particles was comparable to that measured on-line by DMS. A 5–10 wt.% of stable residual was detected for both E4 and E5 soots by termogravimetric analysis (TGA) and indicated the presence of inorganic impurities (additives to the lubricating oil or to the diesel fuel itself, engine wear).

### Exposure to DEP did not affect T cell apoptosis or necrosis

**Figure 1:**
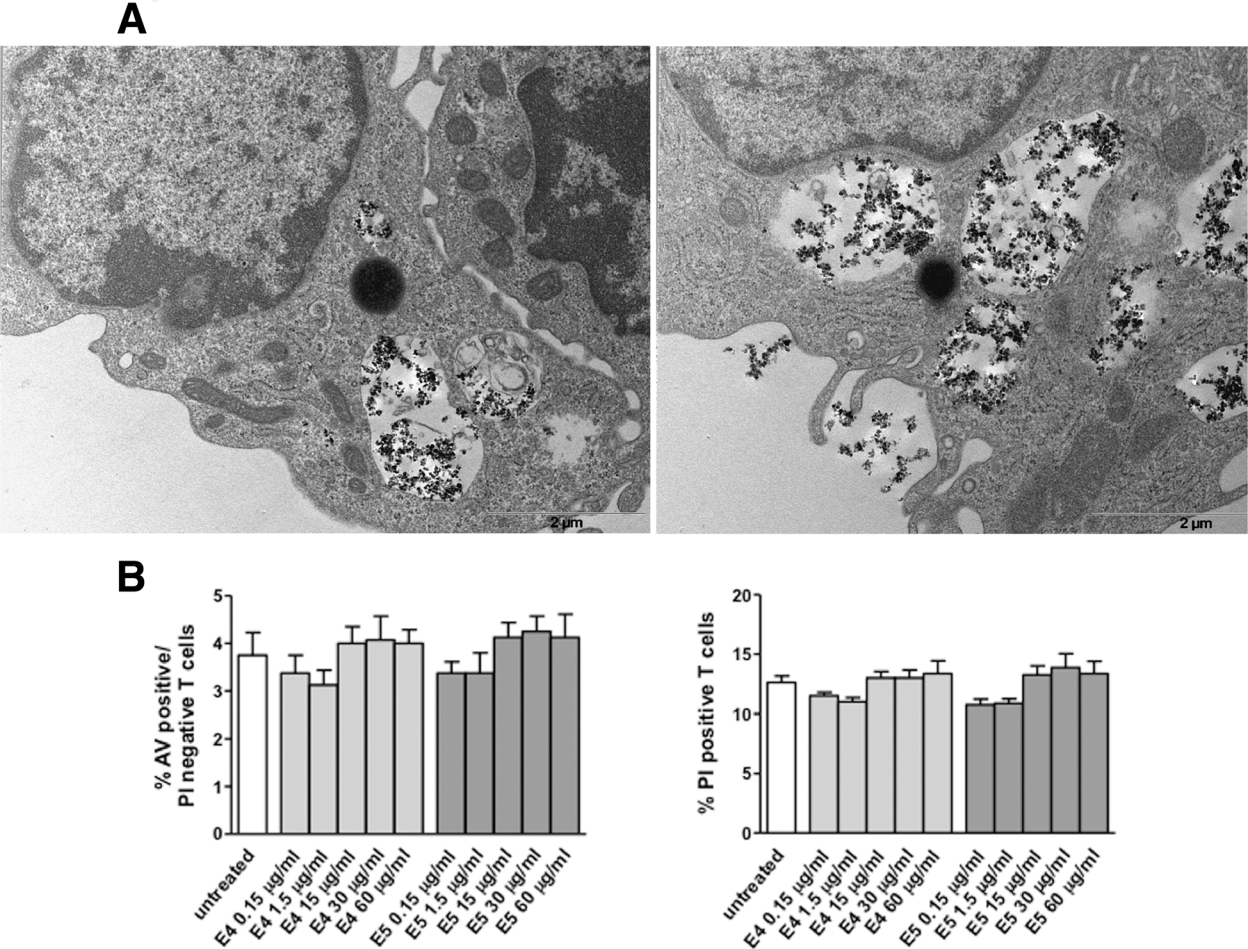
**Uptake of DEP by T lymphocytes and dose–response analysis of apoptosis/necrosis after nanoparticulate exposure. (A)** TEM analysis was performed on T cells after 48 h incubation with E4 or E5 nanoparticles (both used at 30 μg/ml). DEP were found to be localized in membrane-surrounded vesicles in the cytoplasmic region (E4, left panel and E5, right panel). Note the integrity of ultrastructural features of mitochondria and the absence of signs of cell injury. **(B)** Apoptosis/necrosis assay involving dual staining with AV and PI was carried out using flow cytometry. Results of dose–response experiments performed at 48 h are shown. Data referred to both AV positive/PI negative and PI positive T lymphocytes are shown and are presented as mean ± SD of independent experiments performed in cells from 15 healthy donors.

In order to assess the intracellular localization of nanoparticulate in lymphocytes, a transmission electron microscopy (TEM) analysis was carried out. Agglomerates of nanoparticles were found to be incorporated into membrane-bound vacuoles in the cytoplasmic region (Figure 1A: E4, left panel and E5, right panel). No agglomerates of nanoparticles were observed free in the cytoplasm or in the nucleus. No ultrastructural features of cell death, e.g., apoptosis, were detected. Possible changes of apoptosis and/or necrosis levels in response to DEP treatment were further evaluated by using a dual staining with annexin V (AV), a cell surface marker for apoptotic cells and propidium iodide (PI), a DNA intercalating agent which only enters cells that have lost membrane integrity. This assay enables identification of both early (AV positive/PI negative) and late apoptotic or necrotic cells (PI positive). No significant effects on these parameters were observed in T lymphocytes in response to E4 or E5 particles used in the concentration range from 0.15 to 60 μg/ml and at different time-points (i.e., from 24 h to 9 days). Results of dose–response experiments performed at 48 h are shown in Figure 1B. Notably, after 6 and 9 days of culture, a reduction of PI positive T lymphocytes, although not significant (p > 0.05), was detected after DEP treatment (see Additional file 1: Figure S1). At these time points, no changes were observed in treated *versus* untreated cells within the AV positive/PI negative T cell population.

### Exposure to DEP induced autophagic blockade in T lymphocytes

**Figure 2:**
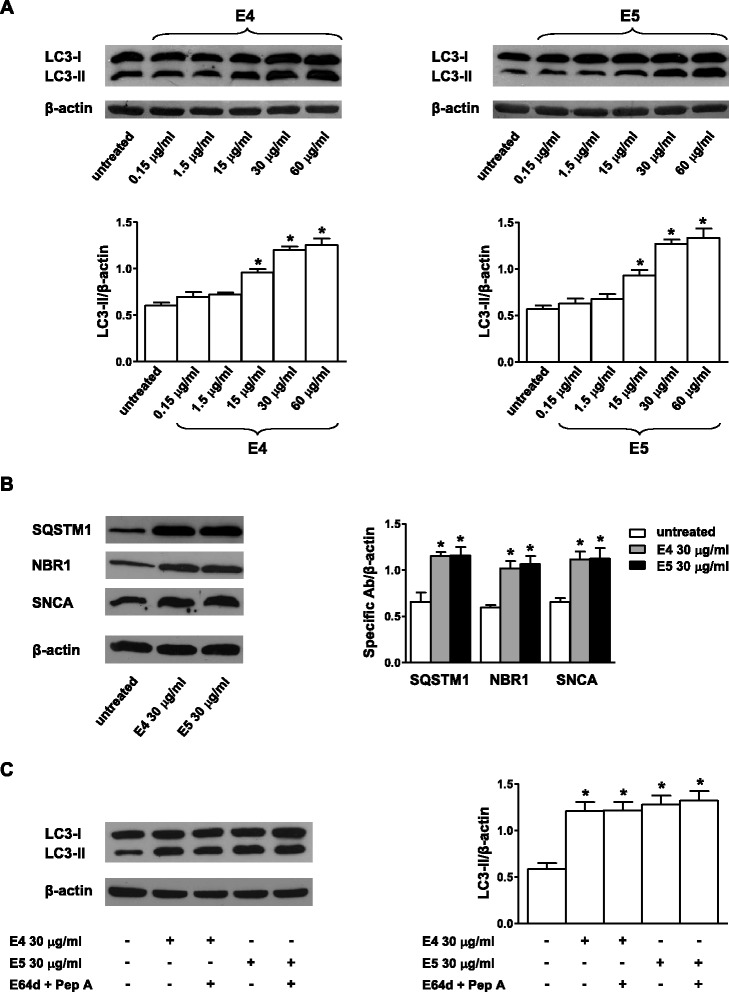
**DEP-induced autophagic-lysosomal blockade in human T lymphocytes. (A)** LC3-II Western blot analysis of T-cell lysates (30 μg/lane) from one representative healthy donor (of the 15 analyzed) after treatment with different concentrations (0.15-60 μg/ml for 48 h) of E4 or E5 particles. Densitometry analysis of LC3-II levels relative to β-actin is also shown. Values are expressed as mean ± SD obtained from independent experiments performed in cells from 15 healthy donors. Statistically significant differences are indicated in the figure. *p *<* 0.05 *versus* untreated cells. **(B)** Western blot analysis of autophagic-lysosomal proteins (SQSTM1, NBR1, SNCA) in T-cell lysates from one representative healthy donor (of the 15 analyzed) after treatment with E4 or E5 (30 μg/ml for 48 h) particles. Densitometry analysis of specific protein levels relative to β-actin is also shown. Values are expressed as mean ± SD obtained from independent experiments performed in cells from 15 healthy donors. Statistically significant differences are indicated in the figure. *p *<* 0.05 *versus* untreated cells. **(C)** LC3-II Western blot analysis of T-cell lysates from one representative healthy donor (of the 15 analyzed) after treatment with E4 or E5 (30 μg/ml for 48 h) particles in the absence or presence of the lysosomal inhibitors E64d and pepstatin A. Densitometry analysis of LC3-II levels relative to β-actin is also shown. Values are expressed as mean ± SD obtained from independent experiments performed in cells from 15 healthy donors. Statistically significant differences are indicated in the figure. *p *<* 0.05 *versus* untreated cells. SQSTM1, sequestosome 1; NBR1, neighbor of BRCA1 gene 1; SNCA, α-synuclein; Pep A, pepstatin A.

Autophagy is detectable in human T lymphocytes and a complex function for it in T lymphocyte development, survival, and proliferation has been recently described [28-31]. During autophagy, portions of cytoplasm are sequestered by double-membrane vesicles, the autophagosomes, and degraded after fusion with lysosomes for subsequent recycling [26]. Here, we investigated whether exposure to DEP could modify the autophagy level in T lymphocytes measuring by Western blot the expression of an established set of autophagosomal markers: microtubule-associated protein 1 light chain 3 (LC3), sequestosome 1 (SQSTM1), neighbor of BRCA1 gene 1 (NBR1), and α-synuclein (SNCA) [37,38]. LC3 (Atg8 in the yeast) is an essential factor for autophagosome formation [39]. Its unlipidated cytosolic form is called LC3-I, whereas the lipidated form is referred as to LC3-II and localizes to autophagosomal membranes throughout the maturation process of the autophagosome. For this reason, LC3-II is commonly used as a specific marker for monitoring autophagy levels [38]. A dose-dependent and time-dependent accumulation of LC3-II occurred in response to treatment with both E4 and E5 particles and a significant increase was observed after 24 h of culture at concentrations starting from 15 μg/ml. Results of dose–response experiments performed at 48 h are shown in Figure 2A. Additionally, a significant accumulation of SQSTM1 and NBR1, substrates that undergo depletion upon autophagy induction [38], was detected (Figure 2B). A similar increase occurred with SNCA (Figure 2B), which is another autophagic substrate protein that accumulates as a consequence of the blockade of autophagic lysosomal flux [37]. Note that in these and the subsequent experiments E4 and E5 particles were used at a concentration of 30 μg/ml for 24 h – 72 h (depending on the studied parameter) on the basis of preliminary dose–response and time-course experiments (see Methods and Additional file 1: Figure S1, for details). In order to gain further insight into the mechanism of DEP-induced autophagic alterations, a LC3 turnover assay, employing the lysosomal inhibitors E64d and pepstatin A (PepA) co-treatment, was performed (Figure 2C). In fact, it is well known that LC3-II can accumulate because of increased upstream autophagosome formation or impaired downstream autophagosome lysosome fusion [38]. To distinguish between these two possibilities, we assayed DEP-induced LC3-II accumulation in the presence or absence of the above mentioned lysosomal protease inhibitors. As observed above, DEP treatment caused an increase of LC3-II levels and, importantly, when DEP exposure occurred in the presence of E64d and PepA, DEP-induced upregulation of LC3-II levels was not potentiated, this being consistent with an autophagic-lysosomal blockade of LC3-II degradation at the autolysosomal level. Previous results by our group [32,33] showed that E4 particles possessed a higher cytotoxic potential as compared to BS particles. Therefore, to compare these compounds in terms of autophagy modulation, we performed the above described set of experiments on BS-treated T lymphocytes. We found that BS induced an autophagic blockade similarly to that observed with E4 and E5 compounds (see Additional file 1: Figure S2).

### Exposure to DEP affected mitochondrial membrane potential (ΔΨm)

**Figure 3:**
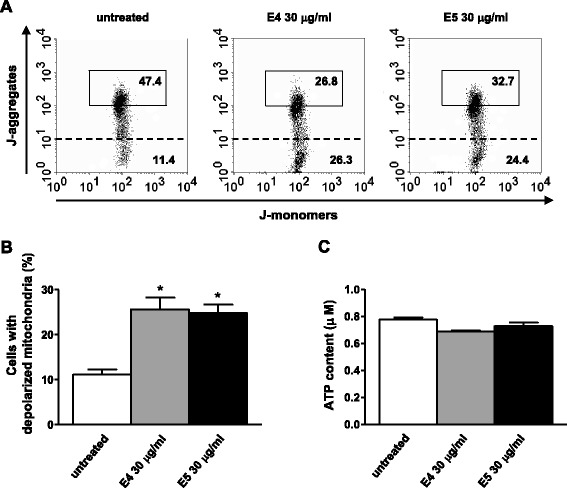
**Loss of ΔΨm but preserved ATP content after exposure of T lymphocytes to DEP. (A)** Flow cytometry analysis of ΔΨm after staining with JC-1 in untreated T lymphocytes (left panel), T lymphocytes treated with E4 (middle panel) or E5 (right panel) particles (30 μg/ml for 24 h for both compounds). The results obtained in a representative experiment are shown. The numbers in the boxed areas represent the percentages of cells with hyperpolarized mitochondria. The percentages of cells with depolarized mitochondria are shown below the dashed line. **(B)** Mean percentage (and SD) of lymphocytes with depolarized mitochondria obtained from independent experiments performed in cells from 15 healthy donors is also shown. *p < 0.05 *versus* untreated cells. **(C)** ATP content detected by chemiluminescent assay in untreated and E4-and E5-treated T lymphocytes (30 μg/ml for 24 h for both compounds). Data are expressed as mean ± SD and are obtained from independent experiments performed in T lymphocytes from 5 out 15 randomly selected healthy donors.

Mitochondria play a primary role in cell physiology, providing the energy supply to the cells as well as controlling their fate [40]. We further characterized DEP cytotoxicity in term of mitochondrial function. To this aim, we first analyzed changes of ∆Ψm in DEP-treated T lymphocytes. Quantitative flow cytometry analysis, performed using the 5,5′,6,6′-tetrachloro-1,1′,3,3′-tetraethylbenzimidazol carbocyanine iodide (JC-1) probe, showed that both E4 and E5 particles induced a significant loss of ∆Ψm already detectable after 24 h of treatment (26 ± 4% and 25 ± 3% respectively *versus* 11 ± 3% of untreated cells, Figure 3A,B). The difference between treated and untreated cells was no longer significant starting from 72 h. To note, loss of ∆Ψm was not followed by an increase in the percentage of apoptotic/necrotic cells that remained unchanged in treated *versus* untreated cells (see above). Because ΔΨm is the driving force for mitochondrial ATP synthesis and loss of ΔΨm may result in depletion of cellular adenosine triphosphate (ATP) level [41], we also measured the ATP content in E4- and E5-treated T lymphocytes. We did not detect any change of this parameter after cell treatment (Figure 3C).

### Exposure to DEP significantly reduced the expression of CD25 molecule but did not interfere with the expression of other T cell activation markers or with proliferation level

**Figure 4:**
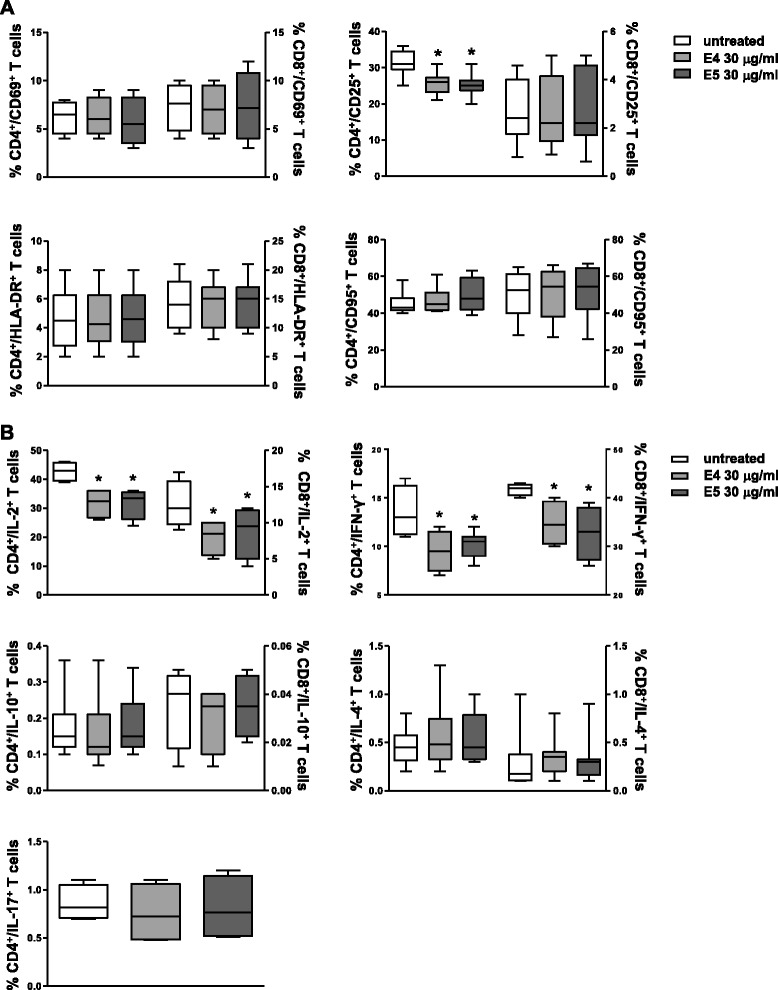
**Flow cytometry immunophenotyping of DEP-treated T lymphocytes.** Flow cytometry analysis of T cell activation markers **(A)** and cytokine expression at the single cell level **(B)** carried out in CD4^+^ and CD8^+^ T lymphocytes from 15 healthy donors after treatment with 30 μg/ml of E4 or E5 particles for 48 h (activation markers) or 72 h (cytokine production). For CD4^+^ and CD8^+^ T lymphocyte subsets, data were expressed as the percentage of each subset within the CD4^+^ or CD8^+^ population considered as 100%. Data are represented as box plots displaying medians, 25^th^ and 75^th^ percentiles as boxes, and 10^th^ and 90^th^ percentiles as whiskers. *p *<* 0.05 *versus* untreated cells.

Next, we examined the possible effects of DEP on the activation state of T lymphocytes as well as on their proliferation rate. To this aim, the expression of activation markers (CD69, CD25, HLA-DR and CD95 molecules) was evaluated in CD4^+^ and CD8^+^ T lymphocytes. The expression of CD25 molecule was down-regulated on CD4^+^, but not on CD8^+^, T cells in response to both E4 and E5 treatments from 24 h to 72 h of cell culture (nadir at 48 h, p = 0.0025 and p = 0.0018 for E4- and E5-treated cells *versus* untreated cells, respectively, Figure 4A) whereas starting from day 6 no differences between untreated and treated cells were detected. Conversely, in the same experimental condition, no changes in the expression of CD69, HLA-DR and CD95 molecules were detected in both CD4^+^ and CD8^+^ T cells (Figure 4A). The effect of exposure to DEP was also evaluated in terms of modulation of T cell proliferation. Both resting and anti-CD3-activated T lymphocytes were treated with E4 or E5 particles and the rate of cell proliferation was detected by measuring the Ki-67 nuclear Ag expression. For T cell activation, both suboptimal (1.25 μg/ml) and optimal (2.5 μg/ml) concentrations of anti-CD3 monoclonal antibody (mAb) were used. As shown in Table 1, exposure to E4 or E5 particles did not have any effect on the level of cell proliferation in both resting and activated T cells.

**Table 1:** **Exposure to DEP did not interfere with T cell proliferation**

	**% Ki-67** ^**+**^ **T lymphocytes**		
	**Untreated**	**E4**	**E5**
Resting T lymphocytes	0.13 ± 0.02	0.12 ± 0.03	0.12 ± 0.03
Activated T lymphocytes:			
anti-CD3 (2.5 μg/ml)	71 ± 5	69 ± 6	67 ± 5
anti-CD3 (1.25 μg/ml)	23 ± 4	22 ± 2	21 ± 3

Data are expressed as mean ± SD and are obtained from independent experiments performed in T cells from 15 healthy donors after cell treatment with E4 or E5 particles (both used at 30 μg/ml for 72 h) in the presence (activated T lymphocytes) or absence (resting T lymphocytes) of anti-CD3 mAb.

### Exposure to DEP significantly reduced Th1 cytokine production

The production of a panel of cytokines, including interleukin (IL)-2, interferon (IFN)-γ, IL-4, IL-10, and IL-17, was evaluated at the single cell level in CD4^+^ and CD8^+^ T cells. Results are summarized in Figure 4B. Exposure to E4 or E5 particles significantly suppressed IL-2 production in CD4^+^ and CD8^+^ T cells without significant differences between the two compounds (CD4^+^ T cells, p < 0.0001 for both E4- and E5-treated cells *versus* untreated cells; CD8^+^ T cells, p = 0.005 and p = 0.034 for E4- and E5-treated cells *versus* untreated cells, respectively). Also for IFN-γ production, after DEP treatment, a significant reduction was observed with both compounds in CD4^+^ (p = 0.003 and p = 0.004 for E4- and E5-treated cells *versus* untreated cells, respectively) and CD8^+^ T cells (p = 0.0005 and p = 0.0002 for E4- or E5-treated cells *versus* untreated cells, respectively). Regarding IL-4, IL-10, and IL-17 production no significant changes were found in treated *versus* untreated cells. In particular, for IL-4 and IL-10 expression level, a great inter-individual variability was detected in response to E4 or E5 particles (Figure 4B).

## Discussion

In this study, we observed that *in vitro* exposure of human T lymphocytes to E4 and E5 diesel exhaust nanoparticles has a strong impact on their phenotype and function. We focused on the role played by the particle core in order to discriminate its effect (not yet reported in the current literature) from that due to the adsorbed species. We also addressed our investigations on the impact of the engine technology level (as the combustion system) on the emitted soot nanoparticles, neglecting the effect of the after-treatment system (diesel oxidation catalyst, DOC, and diesel particulate filter, DPF). It should be noted that although the exhaust after-treatment system changes the physical-chemical features of the raw combustion-formed soot particles, it has been reported that these changes are not dramatic and the nanostructure of the particles that reach the ambient air is strictly correlated to the particles collected upstream the after-treatment system [42].

The efforts of the producers are aimed to further decrease the negative impact on human and animal health of diesel exhaust nanoparticulate reducing particle emission rate as well as introducing filters for soot particles. Since E5 engines emit about a fifth of the E4 engines in terms of mass, their impact, expressed as toxic potential/kilometer or /kWh, is lower. However, our results demonstrate that E5 engines present the same toxic potential of E4 engines in terms of soot quality. These results can be related to the very similar structural features exhibited by the two diesel soots. In particular, the species removed from the soot surface by particle processing are chemically similar in both E4 and E5 soots suggesting that no significant differences in toxicological behavior can be forecasted on the unwashed soot.

To our knowledge, this is the first report describing the effect of DEP on T cell fate in terms of apoptosis, necrosis, and autophagy. Although exposure to E4 or E5 particles does not seem to significantly impact apoptosis or necrosis, it influences the autophagy process inducing an autophagic-lysosomal blockade. Interestingly, a similar effect was observed with carbonaceous particulate from an older diesel engine (i.e., BS), thus suggesting comparable toxicity in terms of autophagy dysfunction between this compound and E4/E5 particles. The defect of autophagosome degradation could be consistent with a functional block induced by DEP at the lysosomal level [43]. In this regard, Chaudhuri *et al.* [44] found that chronic *in vitro* exposure of monocyte-derived macrophages to concentrations of DEP ≥ 10 μg/ml caused a loss of lysosomal acidification and this could result in an impairment of pH control and inactivation of lysosomal proteases. On the other hand, lysosomal overload by nanoparticulate has been proposed as a further mechanism for the blockade of autophagy flux [43]. The finding of an autophagy impairment induced by DEP reveals a critical mechanism by which nanoparticulate could interfere with lymphocyte homeostasis and immune responses. Basal levels of autophagy contribute to the physiological turnover of proteins and to the removal of old and/or damaged organelles [45]. Autophagy is also involved in innate and adaptive immune responses, playing a key role in interactions against microbes [46], in antigen processing for major histocompatibility complex presentation [47], in lymphocyte development, survival, and proliferation [28]. Importantly, over recent years, defective autophagy has been implicated in a number of diseases [45]. For instance, evidence suggests that autophagy blockade can favour cancer development allowing the accumulation of damaged mitochondria that can induce oxidative stress, inflammation and DNA damage [48,49]. Disruption of the autophagy pathway has also been associated with autoimmune disorders such as Systemic Lupus Erythematosus in which autophagy blockade may lead to accumulation of damaged mitochondria, increased production of reactive oxygen species and increased apoptosis, all pathogenetic events in this disease [29,50]. In this context, future studies on affected populations, specifically focused to assess a link between nanoparticulate-induced autophagy dysfunctions and disease development and progression, could provide fruitful information.

Here, we observed that DEP-induced autophagy blockade was concomitant with mitochondrial membrane perturbations. DEP-induced mitochondrial membrane alterations, leading to dissipation of ∆Ψm, have been demonstrated by different studies [51-53] although the biologic consequences of this effect are far from being fully elucidated. Loss of ∆Ψm generally precedes apoptosis [54] and, consistently with this assumption, rat pulmonary alveolar macrophages or murine macrophage cell lines exposed to DEP show an orderly sequence of events, i.e., collapse of ∆Ψm, initiation of apoptosis, uncoupling of oxidative phosphorylation, and decreased ATP production [51,53]. On the other hand, Wang *et al.* [52] found a loss of ∆Ψm in the absence of apoptosis in different human cells (e.g., THP-1 monocytes, A549 lung epithelial cells and primary red blood cells) exposed to DEP. Similar results were obtained by our group in T lymphocytes, suggesting that diesel nanoparticulate has a property that prompts mitochondria membrane collapse without inducing apoptosis. Reduced ∆Ψm and parallel resistance to apoptosis have been described in the mitochondrial DNA-depleted ρ^0^ cells [55] and a depletion of mitochondrial DNA could be hypothesized after DEP exposure. Further research is underway to investigate this issue. Notably, in our experimental conditions, ATP content remained unchanged after DEP treatment suggesting that compensatory mechanism to produce ATP (e.g., glycolysis) could be activated in T lymphocytes to produce a sufficient amount of energy and to maintain housekeeping functions avoiding cell death. Interestingly, as stated above, we observed a reduction of apoptotic cells, although not significant, after 6–9 days of culture. The survival of DEP-treated T lymphocytes could be facilitated by the fact that diesel nanoparticulate seems to favour a quiescent phenotype (e.g., down regulation of CD25 expression) with a low energy demand. Actually, a further mechanism by which DEP could interfere with lymphocyte homeostasis is their immunosuppressive activity. Previously reported data by Mamessier *et al.* [19] showed that DEP-PAH exposure induced the expression of activation markers, including CD25 molecule, on T cells from asthmatic patients but not from controls. Here, we analysed the expression of different cell activation markers separately on CD4^+^ and CD8^+^ T cells from healthy donors and observed that DEP were able to reduce the expression of the CD25 molecule on CD4^+^ T cells. Discrepancies with the data by Mamessier *et al*. [19] could be explained by the different characteristics of the nanoparticulate used (e.g., PAH content) and by the different methodological approach. In fact, our study focused on the effect of DEP on T cells from healthy donors, while T cells from patients affected by chronic respiratory diseases, committed by persistent antigen stimulation to a specific immunological profile [56], were the object of the above mentioned study. Notably, we also found a significant reduction of IL-2 production in both CD4^+^ and CD8^+^ T cells. Interleukin-2 is the prototypic growth factor for T lymphocytes and it promotes T cell survival, proliferation, and differentiation into effector cells [57]. Interleukin-2 also functions to limit immune responses by stimulating the development and functions of regulatory T cells [58] and by promoting Fas-mediated apoptotic death of CD4^+^ T cells [59]. Therefore DEP exposure by decreasing IL-2 production could lead to a defective immune surveillance and to an abnormal persistence of activated T cells. The reduction of IFN-γ production that we observed after DEP exposure in both CD4^+^ and CD8^+^ T cells further contributes to the defective Th1 profile. This finding, in association with the recent observation that DEP decrease markers of cytotoxic natural killer cells and functionally suppress cell-mediated cytotoxicity [60], strongly supports the hypothesis that DEP exposure may increase the susceptibility to viral infections.

## Conclusions

Overall, our data identify some functional and phenotypic T lymphocyte parameters as relevant targets for DEP cytotoxicity, whose impairment could be detrimental, at least in the long run, for human health, favouring the development or the progression of diseases such as cancer and autoimmunity. Further studies are now warranted i) to better elucidate the functional endpoints of DEP actions highlighted by the current study and ii) to address the impact of exhaust after-treatment system on soot nanoparticles during its normal operation and regeneration phase, by collecting the tailpipe emitted particles that represent more strictly the ambient air particulate.

## Methods

### Particle collection and characterization

#### Experimental set up

The experimental activities were conducted on a prototype single cylinder research engine which has a modern combustion system design derived from a E5 compliant four cylinder engine which represents the state of the art of light duty diesel engine technology. The engine out exhaust gases for pollutant and particle analysis were diluted with a ratio of about 8.5, in order to avoid the gas condensation, and sampled at the same point, upstream the typical after treatment systems (DOC and DPF). From the same point the exhaust gases were draw off and collected on a filter. The counting and sizing of particles was performed by means of a DMS (DMS 500, Cambustion, Cambridge, United Kingdom) which measurement principle is based on a deflection of electrically charged particles combined with electrical counting. The DMS 500 uses two internal dilution systems automatically controlled. The first stage was settled at 5:1 while the second one at 200:1, for a total dilution ratio of about 1000:1. The measurement range is from 5 to 1000 nm [61]. The BS soot originated from a D2876 CR engine, operated at 30% load, extra-low rail pressure, and air throttling (blackening number 5), (see Additional file 1 for details).

#### Test methodology

The test procedure and points were chosen in order to provide additional experimental information on soot characteristics. The operating points were performed using E4 and E5 engine calibration (derived from the real four-cylinder engine of equal unit displacement) to ensure the value for practical application in the field of light duty engines. The tests were performed at fixed engine speed (2000 rpm) and load (5 bar brake mean effective pressure). Exhaust gas recirculation rates were set in order to reach the E5 low NO_x_ emissions (26% for E5 and 23% for E4, respectively). Engine conditions were kept equal to the reference E4/5 calibration in both the two test series. A commercial European diesel was used as fuel.

#### Soot sampling and pre-treatment

Total particulate was collected from the exhaust pipe by isokinetic sampling. The sampling line comprised a Teflon filter (pore diameter 0.45 μm, Millipore Corporation, Bradford*,* MA, USA) placed in a temperature controlled system (360 K) to avoid steam condensation. The solid particulate collected on the filter was washed with dichloromethane (DCM) in order to remove condensable species and fuel residuals (soluble organic fraction, SOF). All non-covalently bound molecules (unburned and partially burned fuel, lube oil, PAH), about 15 wt.%, adsorbed on soot surface have been removed by DCM washings. The complete removal of the non-covalently bound molecules was confirmed by infrared spectroscopy (absence of the signals in the wavelength range typical of aliphatic groups) and fluorescence spectroscopy (absence of any detectable fluorescence signal in the DCM after repeated washings). The carbonaceous solid after DCM extraction (soot) was dried, weighted and characterized.

#### Soot characterization

The full chemical-physical characterization of the soot has been performed after washing with DCM in order to probe the soot surface without the interference of physisorbed species (unburned hydrocarbons and tar species). Fourier transform infrared (FTIR) spectra were recorded on a Nicolet iS10 spectrometer (Thermo Fisher Scientific, Rockford*,* IL, USA) using the attenuated total reflectance (ATR) method. The hydrodynamic diameter of the carbonaceous materials was measured by using a Malvern Zetasizer Nano ZS instrument (Malvern Instruments Ltd, Malvern, United Kingdom) on soot suspension in N-methylpyrrolidinone (NMP) [62]. TEM and HRTEM imaging were performed on a FEI Tecnai G2 F20 transmission electron microscope equipped with a field-emission gun (Fei Munich, Gräfelfing, Germany) [34]. Electronic structure measurements were performed using electron energy-loss spectroscopy (EELS).

Ash content was evaluated by TGA performed on a Perkin-Elmer Pyris 1 Thermogravimetric Analyzer in oxidative environment (air, 30 mL min-1). Soot samples were heated from 30°C up to 750°C at a rate of 10°C min-1. UV–vis spectra of soot, suspended in NMP, were acquired on a HP 8453 Diode Array spectrophotometer (Agilent Technologies, Santa Clara, CA, USA). For the *in vitro* studies, the E4 and E5 soots were sterilized by heating at 180°C. Both E4 and E5 soots contained < 0.00025 ng of endotoxin/μg of DEP, as determined by the quantitative chromogenic Limulus amebocyte lysate test (QCL-1000; BioWhittaker, Walkersville, MD, USA). Then the particles were washed three times in distilled water, suspended in phosphate-buffered saline at a stock concentration of 1 mg/ml and sonicated in a water bath at low intensity for 48 h before the use, in order to obtain a better dispersion of the particles that tend to agglomerate.

### Cell purification and culture

Blood samples were obtained from 15 healthy donors (age range, 24–62 years; 7 males and 8 females). Informed consent was obtained from each study participant and the study was approved by the Ethical Committee of “Istituti Fisioterapici Ospedalieri-IFO”, Rome, Italy. All subjects were lifetime nonsmokers and had no history of allergic diseases or chronic respiratory conditions. Peripheral blood mononuclear cells (PBMC) were isolated by Ficoll-Hypaque density-gradient centrifugation. Cells were cultured in RPMI-1640 medium (GIBCO BRL, Grand Island, NY, USA) supplemented with 10% fetal bovine serum (Euroclone, Pero, Milan, Italy), 2 mM glutamine (Sigma, St. Louis, MO, USA) and 50 μg/ml gentamycin (Sigma). Preliminary dose response (0.15, 1.5, 15, 30, and 60 μg/ml) and time course (24, 48 and 72 h and 6 and 9 days) experiments showed that both E4 and E5 particles should be used at a dose of 30 μg/ml and at 24 h – 72 h of culture (depending on the studied parameters) to obtain the highest detectable changes (see Additional file 1: Figure S1). Where indicated, cells were treated in the presence of lysosomal inhibitors E64d and PepA (both at 10 μg/ml; Sigma) for the last 2 h of culture. For T cell proliferation, PBMC were stimulated with coated anti-CD3 mAb (clone UCHT1, 1.25 μg/ml and 2.5 μg/ml, R&D Systems, Minneapolis, MN, USA) for 72 h. Separation of untouched T cells from PBMC was performed by immunomagnetic-based depletion of non T cells using the Pan T Cell isolation Kit II (Miltenyi Biotec, Bergisch-Gladbach, Germany). Purity of isolated cells, assessed by flow cytometer, reached routinely at least 97%.

### Transmission electron microscopy (TEM)

For TEM examination, purified T cells were fixed in 2.5% cacodylate-buffered (0.2 M, pH 7.2) glutaraldehyde for 20 min at room temperature and postfixed in 1% OsO_4_ in cacodylate buffer for 1 h at room temperature. Fixed specimens were dehydrated through a graded series of ethanol solutions and embedded in Agar 100 (Agar Aids, Cambridge, UK). Serial ultrathin sections were collected on 200-mesh grids and then counterstained with uranyl acetate and lead citrate. Sections were observed with a Philips 208 electron microscope at 80 kV.

### Flow cytometry

#### Surface and intracellular phenotyping

Surface and intracellular phenotyping of PBMC was performed with combinations of mAbs fluorescein isothiocyanate (FITC), phycoerythrin (PE), peridinin chlorophyll protein (PerCP), or allophycocyanin (APC) as described before [63]. For surface staining, conjugated mAbs against human CD3, CD4, CD8, CD25, CD95, HLA-DR, CD69, and control mouse IgG1 (all from BD Biosciences, San Jose, CA, USA) were used. Analysis of cytokine production at the single cell level was performed as previously described with minor changes [63]. Briefly, untreated or DEP-treated PBMC were stimulated as follows: i) for IFN-γ, IL-2, and IL-4 analysis, 25 ng/ml phorbol myristate acetate (PMA, Sigma) and 1 μg/ml ionomycin (Sigma) for the last 16 h of culture; ii) for IL-17 analysis, 50 ng/ml PMA (Sigma) and 1 μg/ml ionomycin (Sigma) for the last 4 h of culture; iii) for IL-10, 2.5 μg/ml phytohemagglutinin (Sigma) for the last 16 h of culture. To inhibit cytokine secretion, 10 μg/ml brefeldin A (Sigma) was added to each condition at the beginning of stimulation. Cells were either fixed with 4% paraformaldehyde (PFA) and permeabilized with FACS permeabilizing solution (BD Biosciences) for IFN-γ, IL-2, IL-4, and IL-10 detection or fixed and permeabilized with intracellular fixation and permeabilization buffer (eBioscience, San Diego, CA, USA) for IL-17 detection. The following cytokine-specific mAbs were used: FITC-labeled anti-IFN-γ, FITC-labeled anti-IL-2, PE-labeled anti-IL-4, PE-labeled anti-IL-10 (all from BD Biosciences) and FITC-labeled anti-IL-17A (eBioscience). Surface phenotyping was performed with anti-CD4 APC and anti-CD8 PerCP mAbs (BD Biosciences). Appropriate isotypic negative controls were run in parallel. To determine the frequency of T cell subsets, total lymphocytes were first gated by forward and side scatter and then additionally gated for CD4 or CD8 molecule expression.

#### Apoptosis, ΔΨm, and proliferation

Apoptosis was quantified using a FITC-conjugated AV and PI apoptosis detection kit according to the manufacturer’s protocol (Marine Biological Laboratory, Woods Hole, MA, USA). ∆Ψm was studied by using the lipophilic cationic probe JC-1 (Invitrogen, Carlsbad, CA, USA), as previously described [64]. JC-1 is a metachromatic probe able to enter selectively the mitochondria. It exists in a monomeric form (in the green channel) but, depending on the membrane potential, JC-1 can form aggregates that are associated with a large shift in the emission range (in the orange channel) [65]. JC-1 was dissolved and stored according to the manufacturer’s instructions. In brief, cells were incubated in complete medium for 15 min at 37°C in the dark with 10 μg/ml JC-1 probe. At the end of incubation period cells were analyzed on a flow cytometer. Proliferation was evaluated by measuring the Ki-67 nuclear Ag expression using the PE-mouse anti-human Ki-67 Set according to the manufacturer’s protocol (BD Biosciences).

Acquisition was performed on a FACSCalibur cytometer (BD Biosciences) and 30.000-50.000 events per sample were run. Data were analyzed using the Cell Quest Pro software (BD Biosciences).

### Sodium dodecyl sulfate polyacrylamide gel electrophoresis (SDS-PAGE) and Western blot

Purified T lymphocytes were lysed in RIPA buffer (100 mM Tris–HCl pH 8, 150 mM NaCl, 1% Triton X-100, 1 mM MgCl_2_) in the presence of a complete protease inhibitor mixture (Roche Diagnostics GmbH, Mannheim, Germany). Protein content was determined by the Bradford assay (Bio-Rad Laboratories, Richmond, CA, USA). The samples were loaded onto SDS-PAGE and, after electrophoresis, proteins were transferred onto nitrocellulose membrane (GE Healthcare, Munich, Germany) by means of a Trans-Blot transfer cell (Bio-Rad Laboratories). The membranes were then blocked in 5% nonfat milk for 1 h at room temperature and incubated with the appropriate antibody in Tris-buffered saline (TBS) containing 0.1% Tween 20 and 5% bovine serum albumin or nonfat milk. Regarding SNCA detection, because SNCA monomers tend to easily detach from blotted membranes, resulting in no or very poor detection, after Western blot, nitrocellulose membrane was fixed by incubation for 30 min with TBS containing 0.4% PFA [66]. Rabbit anti-human LC3 (Cell Signaling Technology, Beverly, MA, USA), rabbit anti-human SQSTM1/p62, (Sigma), rabbit anti-human NBR1 (Cell Signaling Technology), and mouse anti-human SNCA (clone syn211, Sigma) were used as primary antibodies. Peroxidase-conjugated goat anti-rabbit IgG (Bio-Rad Laboratories) or anti-mouse IgG (Bio-Rad Laboratories) were used as secondary antibodies and the reactions were developed using the ECL Prime Western Blotting Detection Reagent (GE Healthcare). To ensure the presence of equal amounts of proteins, the membranes were reprobed with a rabbit anti-human β-actin antibody (Sigma). Quantification of protein expression was performed by densitometry analysis of the autoradiograms (GS-700 Imaging Densitometer, Bio-Rad Laboratories).

### Determination of ATP

An equal number of T lymphocytes (2 × 10^5^) per condition was lysed (10 mM Tris pH 7.5, 0.1 M NaCl, 1 mM EDTA, 1% Triton X-100) and ATP production was determined by using a luminescent ATP detection assay kit (Invitrogen) according to the manufacturer’s instructions. A Packard TopCount Microplate Scintillation and Luminescence Counter was used as detection instrument (Packard Instrument Company, Wellesley, MA, USA) and values were calculated based on an ATP standard curve.

### Statistical analysis

Data were analysed using GraphPad Prism v5 (GraphPad Inc., San Diego, CA, USA). The Mann–Whitney unpaired test was used to compare quantitative variables in different treatment groups. p values < 0.05 were considered as significant.

## Additional file

Additional file 1
**Supporting information.**

## Abbreviations

APC: Allophycocyanin
ATP: Adenosine triphosphate
AV: Annexin V
BS: Black soot
DCM: Dichloromethane
DEP: Diesel exhaust particles
DLS: Dynamic light scattering
DMS: Differential mobility spectrometer
DOC: Diesel oxidation catalyst
DPF: Diesel particulate filter
ΔΨm: Mitochondrial membrane potential
E4: Euro 4
E5: Euro 5
FITC: Fluorescein isothiocyanate
HRTEM: High resolution transmission electron microscopy
IFN-γ: Interferon γ
IL: Interleukin
JC-1: 5,5′,6,6′-tetrachloro-1,1′,3,3′-tetraethylbenzimidazol carbocyanine iodide
LC3: Microtubule-associated protein 1 light chain 3
mAb: Monoclonal antibody
NBR1: Neighbor of BRCA1 gene 1
NMP: N-methylpyrrolidinone
PBMC: Peripheral blood mononuclear cells
PAH: Polycyclic aromatic hydrocarbons
PE: Phycoerythrin
PepA: Pepstatin A
PerCP: Peridinin chlorophyll protein
PFA: Paraformaldehyde
PI: Propidium iodide
PMA: Phorbol myristate acetate
SDS-PAGE: Sodium dodecyl sulfate polyacrylamide gel electrophoresis
SNCA: α-synuclein
SQSTM1: Sequestosome 1
TBS: Tris-buffered saline
TEM: Transmission electron microscopy
TGA: Termogravimetric analysis
