# Supplementary material for: Diesel exhaust particle exposure in vitro impacts T lymphocyte phenotype and function
Source: Part Fibre Toxicol. 2014 Dec 14;11:74. doi: 10.1186/s12989-014-0074-0 (PMC4271360; doi:10.1186/s12989-014-0074-0)
Supplement: Additional file 1 — Supporting information. [file 12989_2014_74_MOESM1_ESM.docx]

**Supporting information**

**Supplementary figure S1: Time course of T cell responses to DEP treatment.** Preliminary time course (24, 48 and 72 h and 6 and 9 days) experiments were carried out on T cells from 3 healthy donors. DEP effects on T cell function (i.e., apoptosis, autophagy, mitochondrial membrane potential, proliferation and cytokine production) and phenotype (CD69, CD25, HLA-DR and CD95 expression) were studied using 30 μg/ml of DEP per condition. Changed parameters after DEP treatment are shown. The y axis indicates the ratio between treated *versus* untreated cells. Results showed that both E4 and E5 particles should be used at 24 h – 72 h of culture (depending on the studied parameters, see arrows) to obtain the highest detectable changes with the exception of apoptosis. In fact, differences of apoptosis levels (although not significant) between untreated and treated cells were only detectable at day 6 and 9 of culture.

**Supplementary figure S2: BS-induced autophagic-lysosomal blockade in human T lymphocytes**. **(A)** LC3-II Western blot analysis of T-cell lysates (30 µg/lane) from one representative healthy donor (of the 3 analyzed) after treatment with different concentrations (0.15-60 µg/ml for 48 h) of BS particles. Densitometry analysis of LC3-II levels relative to β-actin is also shown. Values are expressed as mean ± SD obtained from independent experiments performed in cells from 3 healthy donors. Statistically significant differences are indicated in the figure. *, p < 0.05 *versus* untreated cells. A significant increase of LC3-II level was detected starting from a concentration of 0.15 μg/ml **(B)** Western blot analysis of autophagic-lysosomal proteins (SQSTM1, NBR1, SNCA) in T-cell lysates from one representative healthy donor (of the 3 analyzed) after treatment with BS (30 µg/ml for 48 h) particles. Densitometry analysis of specific protein levels relative to β-actin is also shown. Values are expressed as mean ± SD obtained from independent experiments performed in cells from 3 healthy donors. Statistically significant differences are indicated in the figure. *, p < 0.05 versus untreated cells. **(C)** LC3-II Western blot analysis of T-cell lysates from one representative healthy donor (of the 3 analyzed) after treatment with BS (30 µg/ml for 48 h) particles in the absence or presence of the lysosomal inhibitors E64d and pepstatin A. Densitometry analysis of LC3-II levels relative to β-actin is also shown. Values are expressed as mean ± SD obtained from independent experiments performed in cells from 3 healthy donors. Statistically significant differences are indicated in the figure. *, p < 0.05 *versus* untreated cells. SQSTM1, sequestosome 1; NBR1, neighbor of BRCA1 gene 1; SNCA, α-synuclein; Pep A, pepstatin A.

**Methods**

**Black soot (BS) collection and characterization**

The methods of BS soot production and collection have been described elsewhere [1,2]. Briefly, the BS soot originated from a D2876 CR truck engine, operated at 30% load, extra-low rail pressure, and air throttling (blackening number 5). The emission rate of the BS engine is 200 – 600 mg/kWh. The diesel fuel used was a standard low-sulfur type, containing 78% parafﬁn and 22% aromatic hydrocarbons (European Norm 590). The sample was collected directly from the exhaust gas of the engine using a special particle collector that was heated to the exhaust gas temperature at the collection position (200°C). Transmission electron microscopy, energy-dispersive X-ray spectroscopy, and temperature programmed oxidation studies revealed that BS soot did not contain ash from the combusted engine lubricant oil. The mean size of BS soot was 35 nm. BS exhibited a secondary structure consisting of a number of agglomerated spherical particles much larger than the

E4/E5 particles. For the *in vitro* studies, the BS soot were sterilized by heating at 180°C, at a temperature lower than that of the exhaust gas at the collection position (200°C), in order to avoiding to affect the particle properties. Then the particles were washed three times in distilled water, suspended in phosphate buffered saline at a stock concentration of 1mg/ ml and sonicated in a water bath at low intensity for 48 h before the use, in order to obtain a better dispersion of the particles that tend to agglomerate. BS contained < 0.00025 ng of endotoxin/μg of protein, as determined by the quantitative chromogenic Limulus amebocyte lysate test (QCL-1000; BioWhittaker, Walkersville, MD, USA).

**References**

1. Su DS, Serafino A, Muller JO, Jentoft RE, Schlogl R, Fiorito S: **Cytotoxicity and inflammatory potential of soot particles of low-emission diesel engines**. *Environ Sci* *Technol* 2008, **42**:1761-1765.

2. Fiorito S, Mastrofrancesco A, Cardinali G, Rosato E, Salsano F, Su DS, Serafino A, Picardi M: **Effects of carbonaceous nanoparticles from low-emission and older diesel engines on human skin cells**. *Carbon* 2011, **49**:5038-5048.
